# Evaluating the Social Anxiety Depression Levels and Accompanying Psychosocial Problems in Children Diagnosed with Enuresis

**DOI:** 10.7759/cureus.28351

**Published:** 2022-08-24

**Authors:** Zeynep Yılmaz Oztorun, Nalan Gordeles Beser, Kenan Oztorun, Leyla Baysan Arabacı

**Affiliations:** 1 Department of Pediatrics, Omer Halisdemir University School of Medicine, Niğde, TUR; 2 Department of Psychiatric Nursing, Omer Halisdemir University, Zubeyde Hanım School of Health, Niğde, TUR; 3 Department of Urology, Omer Halisdemir University School of Medicine, Niğde, TUR; 4 Department of Psychiatric Nursing, Izmir Katip Celebi University, İzmir, TUR

**Keywords:** psychosocial problems, behavioral treatment, anxiety, depression, monosymptomatic enuresis

## Abstract

Introduction: This study was conducted to evaluate the accompanying psychosocial problems in enuresis as well as the social anxiety-depression levels in children diagnosed with enuresis.

Methods: This descriptive study investigating depression and social anxiety levels of children diagnosed with enuresis was carried out with 167 children who were followed up for at least six months following their enuresis diagnosis. All participating children had no other physical or mental illnesses and were aged five years or older. Data were collected using three measurement tools and evaluated using descriptive, correlational analyzes.

Results: In this study, 69.4% of the children diagnosed with enuresis were aged 7-12 years. 38.3% of their parents used “reward”, while 37.1% preferred “punishment” as a method for toilet training their children. The children’s depression and social anxiety mean scores were 21.48±7.29 and 54.58±8.84, respectively. A strong positive correlation was found between the children’s mean depression and social anxiety scores (r=0.677, p<0.001). The median depression scores of children were found to be statistically significantly different according to the children’s type of family, night sleep characteristics, type of enuresis, school success, and family life (p<0.05). Those with fragmented families (p=0.049), who did not sleep deeply at night (p=0.031), who slept for about 5-7 hours a day (p<0.01), and those whose school success was negatively affected by enuresis (p=0.021) and those who were exposed to negative family life (p=0.034) all obtained statistically significantly higher median depression scores.

Conclusion: Children with enuresis had low depression and moderate social anxiety and their depression increased as their social anxiety increased. The children experienced psychosocial difficulties due to enuresis. When treating children with enuresis, it is necessary to consider both the affected child and their families through the adoption of a holistic approach, and also to evaluate the children both physically and psychosocially.

## Introduction

Enuresis negatively affects the lives of both children and their families both physically and psychologically [1]. Enuresis without other low urinary tract symptoms (LUTS) and bladder dysfunction is defined as monosymptomatic enuresis. Children with enuresis and any LUTS are said to have non-monosymptomatic enuresis. Enuresis is subdivided according to its onset, including secondary enuresis that is reserved for children who have had a previous dry period >6 months, otherwise, it is called primary enuresis. Monosymptomatic enuresis refers to involuntary leakage of urine during sleep in children, in the absence of a congenital or acquired central nervous system or urological defects, and without daytime incontinence [2]. The actual cause of monosymptomatic enuresis is still unclear and it is a sociomedical problem that can last into adulthood [3]. Turkish studies report an association between low educational levels among family members and enuresis in children [4,5].

Monosymptomatic enuresis negatively affects children’s physical development and self-esteem, causing shame and mental-health deterioration [1,2]. Treatment approaches to children with enuresis should target overcoming the disease without damaging the child’s sense of self, while any accompanying problems should be dealt with by their families and treatment team members. Physicians involved in the treatment of children with enuresis and nurses responsible for their care should give information to the affected children about the causes of enuresis, as well as how to cope with accompanying problems and responsibilities in the treatment process. [6-8]. Among the treatment recommendations, fluid restriction from 8 pm is reported. Furthermore, relevant training should be given to the child’s parents regarding their treatment roles, and the positive parental attitudes they should adopt toward their child [7,8].

We hypothesized that negative family life and school success can be affected by enuresis at night and the median depression score of children can vary according to night sleep characteristics and type of enuresis. Increased social anxiety may be related to depression [9].

We aimed to examine the psychosocial problems accompanying enuresis and social anxiety-depression levels in children with enuresis who had been referred to pediatrics and urology outpatient clinics of an academic hospital.

## Materials and methods

Written informed consent was obtained from all caregivers of the participants in the study. The study protocol was approved by the Omer Halisdemir University local ethics committee (2018/12). This cross-sectional and descriptive study was conducted to examine the causes of enuresis, depression, and social anxiety levels of children diagnosed with enuresis. This study was carried out between May 21, 2018 and May 20, 2019. The study involved 167 children who were referred to the pediatric and urology outpatient clinics of an academic hospital. All children were followed up for at least six months after their enuresis diagnosis; they had no other physical or mental illness and were aged five years or older; they agreed to participate in the study, and had parents who also agreed to participate in this study. Those who received anxiolytic therapy were excluded from the study because it would affect the anxiety scale. Data were collected using an Introductory Information Form (IIF), the Children’s Depression Inventory (CDI), and the Social Anxiety Scale for Children-Revised (SASC-R) (Refer appendices).

IIF

The form comprises 30 questions, to which the child, the child’s accompanying parents, and the researcher physician have to respond. The questions concern the sociodemographic characteristics of children and their families, as well as the causes and frequency of enuresis. We used bladder diaries and voiding frequency to rule out organic causes of enuresis and we used an ultrasound imaging test to rule out urologic abnormalities. During the follow-up period, we ruled out urinary tract infections by urine culture.

CDI

This scale was developed by Kovacs (1981) to assess depression in children. The scale was adapted to the Turkish language by Öy (1991). The CDI is a 27-item self-assessment scale that can be applied to children aged 6-17 years. Each item is scored as 0, 1, or 2 depending on the severity of the symptom. The maximum score is 54, and a higher score indicates a higher depressive state. Öy (1991) reported the test-retest reliability and internal consistency coefficients of the scale to be 0.70 and 0.80, respectively [10]. In this study, the reliability value of the scale was found to be 0.810.

SASC-R

This five-point Likert-type scale, which is based on self-reported answers, was developed by La Greca et al. [11]. The scale was adapted to the Turkish language by Demir et al. (2000) [12]. The Cronbach’s alpha internal consistency coefficient of the SASC-R was 0.81, and the scale was found to have a high test-retest reliability (r=0.81). The validity study of the scale, which was performed using social phobia patients referred to the clinic, could be used to distinguish these patients from normal, control patients. The lowest and highest scores possible from the scale are 18 and 90, respectively [12]. In this study, the reliability value of the scale was found to be 0.657.

Data were evaluated using descriptive statistics. The Mann-Whitney U test and Kruskal-Wallis test were used for nonparametric assumptions when comparing the scores of social anxiety and depression in enuresis. While evaluating qualitative data, Pearson's chi-square test, Fisher's exact chi-square test, and Yates correction test were used. Pearson's correlation coefficient test was used to find the relationship between two scale scores.

## Results

In this study, 190 children were invited to the research. However, 167 children accepted to join the research. 69.4% of the children participants who had enuresis were aged 7-12 years, 74.2% lived in a city center, and 66.4% were from families that had a middle-income level. 71.3% of the children’s mothers were housewives and 90.4% of their fathers were employed (Table 1).

**Table 1:** Children’s Socio-Demographic Characteristics

SOCIO-DEMOGRAPHICS	Number				%		
AGE							
5–6 years	29				17.4		
7–12 years	116				69.4		
13 years	22				13.2		
GENDER							
Female	99		59.3				
Male	68		40.7				
SCHOOL ATTENDANCE							
Yes	163				97.6		
No	4				2.4		
INCOME LEVEL							
Good	23				13.8		
Middle	111				66.4		
Poor	33				19.8		
LIVING PLACE							
Village/town	22				13.2		
County	21				12.6		
City	124				74.2		
TYPE OF FAMILY							
Nuclear	150				89.8		
Extended	14				8.4		
Fragmented	3				1.8		
MOTHER’S EDUCATION LEVEL							
Literate	20				12.0		
Secondary school	72				43.1		
High school	71				42.5		
University-college	4				2.4		
FATHER’S EDUCATION LEVEL							
Illiterate	2				1.2		
Primary school	21				12.6		
Secondary school	15				9.0		
High school	103				61.6		
University-college	26				15.6		
MOTHER’S JOB							
Housewife	119				71.3		
Officer	29				17.4		
Worker	16				9.6		
Self-employed	3				1.7		
FATHER’S JOB							
Unemployed	5			3.0			
Officer	30			18.0			
Worker	88			52.7			
Retired	11			6.6			
Self-employed	33			19.7			

Of the children, 87.4% had not experienced negativity in family relationships, 80.2% had deep sleep at night, 80.8% drank water before bedtime, and 82.6% drank 5-6 glasses of water per day; 60% of children have a history of enuresis in at least one parent. Concerning toilet training, 38.3% of the children’s parents used “reward” and 37.1% used “punishment” methods; 96.4% had been treated for 0-6 months (Table 2).

**Table 2:** Factors that May be Related to Enuresis

		Number	%
	Negativity in Family Relations		
	Yes	21	12.6
	No	146	87.4
	Any Change in Family Life		
	Yes	14	8.4
	No	153	91.6
Deep Sleeping Status			
	Deep sleepers	134	80.2
	Light sleepers	33	19.8
Daytime Sleep			
	Yes	6	3.6
	No	161	96.4
Sleep at night			
	5–7 hours	79	47.3
	8–10 hours	88	52.7
Water Intake Before Bedtime			
	Yes	135	80.8
	No	32	19.2
Daily Water Intake			
	5–6 glasses	138	82.6
	7–8 glasses	24	14.4
	1-3 times a week bedwetting	28	16.7
	0-6months treatment time	161	96.4
	7 months-1 year treatment time	6	3.6
	Toilet Training Method Applied by the Family		
	Reward	64	38.3
	Penalty	62	37.1
	Intimidation	23	13.8
	Threat	8	4.8
	No method	10	6.0

Regarding the psychosocial problems the children had due to enuresis, all children reported that they felt “bad”, both on the day they wet the bed/clothes, and on the day (if any) that their friends or relatives learned about it. In addition, 70.1% of children reported that it negatively affected communication with their friends at school, and 82.0% reported that it negatively affected school performance when they wet the bed/clothes. Furthermore, 53.3% stated that they were unable to sleep due to the fear of bedwetting (Table 3).

**Table 3:** Distribution of Children According to Problems Experienced After Bedwetting

Problems Experienced after Bedwetting		Number	%
Feelings on the day of bedwetting			
Feeling bad		167	100.0
Effect of bedwetting on school friendships			
	Negatively affected	117	70.1
	Not affected	50	29.9
Effect of bedwetting on school success			
	Negatively affected	137	82.0
	Not affected	30	18.0
Feelings of children when friends learn about their bedwetting issue			
	Feeling bad	167	100.0
	Feelings of children when relatives learn about their bedwetting issue		
	Feeling bad	167	100.0
	Unwillingness to fall asleep due to fear of bedwetting		
	Yes	89	53.3
	No	78	46.7

The children’s mean depression and social anxiety scores were 21.48±7.29 and 54.58±8.84, respectively. A statistically significant positive correlation was found between their depression and social anxiety mean scores (r=0.677, p<0.001). No statistically significant difference was found between the children’s median social anxiety scores according to their sociodemographic characteristics, causes of enuresis, diagnosis of children, enuresis-related features, and negative feelings due to enuresis (p>0.05).

The median depression scores of children were found to be statistically significantly different according to the children’s type of family, night sleep characteristics, type of enuresis, school success, and family life (p<0.05). Those with fragmented families (p=0.049), who did not sleep deeply at night (p=0.031), who slept for about 5-7 hours a day (p<0.01), those whose school success was negatively affected by enuresis (p=0.021) and those who were exposed to negative family life (p=0.034) all obtained statistically significantly higher median depression scores (Table 4).

**Table 4:** Distribution of Points of Children’s Depression Inventory and Social Anxiety Scale Related to Children Characteristics

	Children’s Depression Inventory			Social Anxiety Scale Related To Children		
	Mean	Median	Min-Max	Mean	Median	Min-Max
Type of Family						
Nuclear	21.22±7.31	23.29	0-32	54.00±8.21	55.34	27-86
Extended	22.07±6.14	23.00	10-30	58.71±13.16	57.33	41-95
Fragmented	31.66±5.13	33.00	26-36	64.33±7.63	66.00	56-71
	p=0.049 p<0.05			p=0.074 p>0.05		
Treatment Time						
0–6 months	21.47±7.21	23.31	0-36	54.70±8.88	55.70	27-95
7 months–1 year	21.66±9.99	25.50	3-29	51.16±7.62	54.33	36-56
	p=0.507			p=0.176		
Frequency of bedwetting						
1–3 times a week	20.39±8.43	22.83	1-36	55.39±9.88	54.42	30-73
4–7 times a week	21.70±7.05	23.50	0-33	54.82±8.64	5.84	27-95
	p=0.450			p=0.311		
Sleep at night						
5–7 hours	23.97±4.65	24.78	10-32	56.27±7.16	55.87	38-95
8–10 hours	19.25±8.45	22.09	0-36	53.05±9.91	55.21	27-86
	p<0.01			p=0.138		
Deep Sleeping Status						
Deep sleepers	20.81±7,63	21.11	0-36	54.24±9.59	55.46	27-95
Light sleepers	24.21±4.93	25.11	7-32	55.93±4.58	56.12	44-67
	p=0.031			p=0.428		
Effect of bedwetting on school success						
Negatively affected	22.15±6.79	23.85	0-36	18.43±8.74	21.75	2-32
Not affected	55.03±8.84	55.70	30-95	52.50±8.70	55.00	27-62
	p=0.021			p=0.347		

The prevalence of enuresis among the children did not differ by a statistically significant amount according to night-sleep time and fluid intake before bedtime (p>0.05); however, a statistically significant difference was seen in children with negativity in their familial life, and toilet training method (p<0.05). The majority of children who wet the bed 4-7 times a week were not exposed to negative family life (χ2=9.634, p=0.002), and had parents who did not use a “punishment” method for toilet training (χ2=9.608, p=0.048).

## Discussion

This study was conducted to examine depression and social anxiety levels of children diagnosed with enuresis. More than half of the children who participated in this study attended school, lived in a city center together with their parents and/or siblings, and had a nuclear family with a middle-income level. The majority of the participating children’s mothers were secondary school graduates and housewives, whereas the majority of the participating children’s fathers were high school graduates and employed.

No studies have yet been found on the school attendance of children diagnosed with enuresis. However, in their study, Güneş et al. report that the majority of children diagnosed with enuresis live in villages and have low-income levels [13]. Studies conducted in different regions of Turkey report that children diagnosed with enuresis have parents with low educational levels [4,14]. Furthermore, Gür et al. argue that enuresis is more common in children with unemployed mothers [4]. Although these results suggest that parents’ educational level, professional characteristics, and income status do not directly affect the state of enuresis in children, these variables are considered to indirectly affect their approach to both the causes and situations of enuresis (hygiene, urinary infection, parasite, etc.), as well as their knowledge, skills, and attitudes in managing these situations.

The majority of the children who participated in this study had no diseases or accidents during or after birth and were not exposed to physical or psychosocial changes in family life that might have caused enuresis. The large majority of participating children slept at any time during the day, drank 5-6 glasses of water before bedtime, and slept deeply at night. In addition, most of them had enuresis symptoms, including wetting the bed/clothes 4-7 times a week and urinating seven times or more a day. Similarly, studies state that hygiene deficiency, family history of enuresis, and deep sleep at night are among the factors related to enuresis [15]. In addition, studies emphasize that the toilet training attitudes of parents affect the development of enuresis in children. By supporting existing studies in the literature suggesting that punitive toilet-training methods are associated with the development of enuresis in children [16], this study found that the majority of the parents of participating children who reported wetting the bed/clothes 4-7 times a week used punishment as a toilet-training method and that this difference was statistically significant (p<0.05). This study also determined that the prevalence of bedwetting at night varies significantly according to the toilet training methods used by the child’s parents. Accordingly, children who receive toilet training with punitive methods wet the bed/clothes 4-7 times a week. In addition, children who do not have a specific urination pattern/habit and those who do not have a family history of any adverse events wet the bed/clothes 4-7 times a week. Accordingly, oppressive and/or inconsistent/extremely flexible parental attitudes are considered to affect the development of enuresis in children.

It was found that the children’s mean depression scale score was below average and that their social anxiety mean score was of a moderate level. In addition, a strong positive correlation was found between their social anxiety and depression scores (p<0.001); that is, as their social anxiety increases, their tendency to depression increases. In fact, all children reported that they felt “bad”, both on the day they the wet bed/clothes and on the day on which their friends/relatives learned about it. In addition, most of them reported having negatively affected school performance and communication with school friends after wetting the bed/clothes, in addition to stating that they were unable to sleep due to the fear of bedwetting.

Eray et al. suggest that adolescents diagnosed with primary monosymptomatic enuresis feel overly distressed in social environments in which they are involved for the first time and that they often avoid social and public areas [17]. And another study by Keten et al. reported higher mean depression scale scores of children-adolescents with primary enuresis (25.0±3.5) as well as lower mean social anxiety score (440±13.6) when compared with the children included in the present study [18]. They also reported that children with primary enuresis and those in the control group obtained the same mean depression score, but that their mean social anxiety score was significantly higher for the control group [19]. In their study, Van Hoecke et al. determined that both children with enuresis and those in a control group obtained similar social anxiety and depression scores according to the forms filled out by children, but that the children with enuresis were significantly anxious and depressive according to the forms (the Behavior Assessment System for Children) filled out by their parents [19]. This difference between the studies may be due to the different measurement tools, research methods, and sample groups used in these studies. Despite differences in their results, one expected result is that children diagnosed with enuresis display social anxiety and depression symptoms. Therefore, it should be noted that physical examination and evaluation of these children alone will be insufficient, and so a holistic psychosocial assessment is both necessary and important for the permanence and continuity of their treatment. Supporting these results, this study found that children with fragmented families, those unable to sleep deeply at night, those who sleep for 5-7 hours a day, those whose school success is negatively affected by enuresis, and those who are exposed to negative family life are more likely to get depression.

Similarly, Hjalmas et al. found that enuretic children were more excited, impatient, irritable, and restless when compared with healthy children [20]. İşcan et al. determined that children with nocturnal enuresis have impaired emotional well-being, self-esteem, and quality of life, thereby affecting their relationships with family and friends [21]. Joinson et al. compared enuretic and healthy children and found a higher number of psychological problems in children with enuresis [22].

The small number of patients included in the study is one of the limitations of the study. The absence of a control group in the study is another limitation of the study. Since almost all children had been treated for enuresis, this may have affected the outcome of the CDI and SASC scores. As the socio-cultural and intellectual levels of the parents were not similar to each other, the perception of the survey questions may not have been sufficiently understood.

## Conclusions

Children who participated in this study stated that they did not experience any physical or psychosocial changes. any disease, accident, etc. that would cause enuresis in their past or present individual and family life. However, they showed low depression and moderate social-anxiety symptoms, and it was found that their depression increased as their social anxiety increased. In addition, children with enuresis experience certain psychosocial difficulties. In line with these results, it is necessary and important to consider not only the affected children but also their families by adopting a holistic treatment approach and evaluating the children both physically and psychosocially. We find that a negative parental attitude influences a child's experiences with bedwetting. Behavioral treatment approaches are recommended rather than medical treatment to reduce the level of anxiety in children with enuresis. Future studies are required to compare children within nuclear families: the enuretic versus non-enuretic.

Parents of children with enuresis should be provided with training on child development and toilet training in consideration of their socioeconomic and sociocultural characteristics. Education and providing good information to parents and the environment are paramount.

## Appendices

PATIENT QUESTIONNAIRE

Below are questions prepared to learn some information about you and your disease. Your answers to the questions will be kept strictly confidential. Thank you for your participation.

SECTION TO ANSWER - PARENT AND CHILD TOGETHER

Child age…….

Child sex                             a. Female             b. Male

Child's attendance to daycare/nursery/school?

a) Yes………….  b.) No ……….. 

Family economic situation?

a.Very well     b. Good   c. Medium    d. Bad    e. Very bad

5.       Indicate the place of residence where the family has lived the longest?

a.     Village /town     b. City      c) Abroad

Family type:

a.     Nuclear family  b.Extended family              c. Broken family

Whom does your child live with at home? ……

Mother’s education type:

a)      illiterate

b)      literate

c)      primary school graduate

d)      secondary school graduate

e)      high school graduate

f)       university graduate

Father’s education type:

a)      illiterate

b)      literate

c)      primary school graduate

d)      secondary school graduate

e)      high school graduate

f)       university graduate     

What is the mother’s job

a)      house wife

b)     officer

c)      employee

d)    retarded

e)     self-employment

f)       Other……

What is the father’s job?

a)    unemployed

b)     officer

c)    employee

d)    retarded

e)   self-employment

f)     Other…… 

12. Was there any problem in the birth of the child? 

        a. Yes     b. No

13. The presence of an important disease or accident that the child has suffered (urinary tract infection, allergy, parasite, accident causing head trauma, etc.)?

     a. Yes        b. No

14. Child's sleep habits (Please answer the following questions):

 Deep sleep?……………………                  a. Yes         b.  No

 Daytime sleepiness? …………..             a. Yes        b.  No

 How many hours does she sleep at night?       a. Yes        b. No

 Fluid consumption 1-2 hours before bedtime … a. Yes   b.  No

The amount of fluid consumed per day ………………………..

15. Frequency of child wetting ………………(Day/week-how much ).

16. The time of bedwetting :          a. Day      b. Night   c. Both day and night

17. At what times and during which activity does the child's bedwetting time usually   occur?………………………………………………………

18. The child's urination habits and problems:

         Burning, pain, etc. when urinating?        a. Yes                        b. No 

         Frequency of urination of the child………………..(per day)

         Is there urgency of the child?    a. Yes      b. No

        Urination type of the child? ………………( urinary retention maneuver, drip urination)

19. Has the child constipation?

a. Yes…….The frequency of defecation              b.  No

20. Negative events experienced in the family and other individuals with whom they have close relationships (divorce, death, chronic illness…) ?

                  a. Yes………..(which of them)    b. No

21. The occurrence of any event that causes a change in family life (a new birth of a sibling, one of the older siblings leaving home, one of the parents leaving home for any reason, etc)?

a. Yes………..                             b. No

22. The method used by the parents during the toilet habit of the child (reward/punishment/frightened/threatening)

 23. The time the child was treated with a diagnosis of enuresis ………………..

THE CHILD SHOULD ANSWER (complete the sentences)

 27. I feel……………..when I have bedwetting 

 28. Bed wetting ………………………….with my friends at school.

 29. Bedwetting felt  my school success……………………….

 30. I…………..my friends learn my bedwetting. If they learn it, I feel……………

 31. I ………………………… my relatives, brothers, cousins ​​to hear that I wet my bed 

        I feel myself …………………………..when they hear it.

32. I don’t sleep comfortably because of the feeling of bedwetting.

             a.True                          b. Wrong

DEPRESSION SCALE FOR CHILDREN

1.   0. I don't feel sad .

      1.  I feel sad  sometimes

      2. I feel all the time.

2     0.. My affairs will never go well

        1. I'm not sure if things will work out.

        2.  My business will go well..

3.       0. I make most of my work right.

           1. I do most of my work wrong.

           2  I do everything wrong

4.       0. I like many things. 

           1. I like some things.

           2.  I don’t like anything.

5        0. I am always a bad child.

          1.  I am a mostly bad child. 

          2.  I am sometimes a bad child.     

6.      0. Every now and then, I think something bad will happen to me.

         1.  I often worry that bad things will happen to me.

        2.   I worry that very bad things will happen to me.

7.     0.  I hate myself.

        1.  I don't like myself.

        2.  I like myself.

8      0.  All bad things are my fault

        1. Some of the bad things are my fault.

         2.  Bad things are not usually my fault.

9.      0. I don't think about killing myself

         1. I think about killing myself but I can't

        2.  I think about killing myself.

10   0. I feel like crying every day.

       1.  Many days I feel like crying.

       2.  Every now and then I feel like crying

11   0. Everything always bores me

       1.  Everything often bores me

       2.   Everything bores me sometimes.

12.    0.  I like to be with people

         1.  I don't like being with people most of the time.

         2.  I never like to be with people.

13     0.  I can't decide on anything.

         1.   It's hard to make a decision about anything.

        2.   I can easily make decisions about anything.

14.   0.  I'm beautiful/handsome

        1.    I have bad parts

        2.    I am ugly.

15   0.  I always push myself to do my school homework.

       1.    I always push myself to do my school homework.

       2.    It's okay to do my schoolwork

16    0. I have trouble sleeping every night

         1. I have trouble sleeping many nights.

         2   I sleep pretty well.

17.   0. I feel tired every now and then 

         1.   I feel tired many days.

        2.  I feel tired always. 

18.     0.  Almost every day, I don't want to eat.

         1.   Most days I don't want to eat.

          2.   I eat pretty well.

19.    0.  I don’t worry about pain.

         1.   I worry about pain mostly.

         2.    I worry about pain always. 

20.    0.  I feel alone.

         1.    I feel alone many times.

         2.   I feel alone always.

21     0.  I don’t like school.

         1.    I like school sometimes.

         2.    I like school many times.

22.    0.  I have a lot of friends.

         1.  I have a few friends but I would like to have more. 

         2.    I have no friends.

23.    0. My school performance is well.

         1.  My school performance is not as good as before.

         2.   I'm failing a lot of lessons I used to be good at.

24     0.  I am not well like the other children.

         1.    If I want I can be well like the other children

        2.   I am well like the other children.

25     0.  Nobody likes me.

         1.   I am not sure if anyone likes me.

        2.     I am sure that somebody likes me

26.    0.  I usually do what I'm told.

         1.   I never do what I'm told.

27.     0. I get along well with people.

          1.  I often fight with people.

          2.  I always fight with people.

SOCIAL ANXIETY SCALE FOR CHILDREN 

Give that sentence a score between 1 and 5 according to the relevance of the sentence to you 

1= never         2= slightly       3= sometimes                 4= mostly            5= always

1.         I'm uncomfortable doing something new in front of other kids.

2           I'm sick of being made fun of

3.         I feel ashamed in front of children I don't know

4.         I think other kids are talking behind my back

5.         I only talk to kids I know well

6.         I worry about what other kids think of me.

7.         I'm afraid other kids won't like me

8.         I feel uncomfortable talking to children I don't know well

9.         I worry about what other kids will say about me.

10.     I feel uncomfortable talking to children I have just met

11.     I'm upset that other kids don't like me

12.     I stay quiet when I'm with a group of kids

13.     I think the other kids are making fun of me

14.     I'm afraid if I argue with another boy, he won't like me

15.     I hesitate to invite others to my house because they may say no.

16.     I get uncomfortable around some children

17      I feel embarrassed even around children I know well.

18.     It's hard for me to offer other kids to play together

## References

[1] Ellington EE, McGuinness TM (2012). Mental health considerations in pediatric enuresis. J Psychosoc Nurs Ment Health Serv.

[2] Austin PF, Bauer SB, Bower W (2016). The standardization of terminology of lower urinary tract function in children and adolescents: update report from the standardization committee of the International Children's Continence Society. Neurourol Urodyn.

[3] Moursy EE, Kamel NF, Kaseem AF (2014). Combined laser acupuncture and desmopressin for treating resistant cases of monosymptomatic nocturnal enuresis: a randomized comparative study. Scand J Urol.

[4] Gür E, Turhan P, Can G (2004). Enuresis: prevalence, risk factors and urinary pathology among school children in Istanbul, Turkey. Pediatr Int.

[5] Ozden C, Ozdal OL, Altinova S, Oguzulgen I, Urgancioglu G, Memis A (2007). Prevalence and associated factors of enuresis in Turkish children. Int Braz J Urol.

[6] Bogaert G, Stein R, Undre S (2020). Practical recommendations of the EAU-ESPU guidelines committee for monosymptomatic enuresis-Bedwetting. Neurourol Urodyn.

[7] Caldwell PH, Nankivell G, Sureshkumar P (2013). Simple behavioural interventions for nocturnal enuresis in children. Cochrane Database Syst Rev.

[8] Glazener CM, Evans JH, Peto RE (2004). Complex behavioural and educational interventions for nocturnal enuresis in children. Cochrane Database Syst Rev.

[9] Gunlicks-Stoessel M, Westervelt A, Reigstad K, Mufson L, Lee S (2019). The role of attachment style in interpersonal psychotherapy for depressed adolescents. Psychother Res.

[10] Öy B (1991). Çocuklar için depresyon ölçeği: Geçerlik ve güvenirlik çalışması [Article in Turkish]. Türk Psikiyatri Dergisi.

[11] Ginsburg GS, La Greca AM, Silverman WK (1998). Social anxiety in children with anxiety disorders: relation with social and emotional functioning. J Abnorm Child Psychol.

[12] Demir T, Eralp-Demir D, Türksoy N (2000). Çocuklar için sosyal anksiyete ölçeğinin geçerlilik ve güvenilirliği [Article in Turkish]. Düşünen Adam Dergisi.

[13] Gunes A, Gunes G, Acik Y, Akilli A (2009). The epidemiology and factors associated with nocturnal enuresis among boarding and daytime school children in southeast of Turkey: a cross sectional study. BMC Public Health.

[14] Gümüş B, Vurgun N, Lekili M, Işcan A, Müezzinoğlu T, Büyuksu C (1999). Prevalence of nocturnal enuresis and accompanying factors in children aged 7-11 years in Turkey. Acta Paediatr.

[15] Walker RA (2019). Nocturnal Enuresis. Prim Care.

[16] Ferrara P, Di Giuseppe M, Fabrizio GC, Sbordone A, Amato M, Cutrona C, Verrotti A (2016). Enuresis and punishment: the adverse effects on child development and on treatment. Urol Int.

[17] Eray Ş, Tekcan D, Baran Y (2019). More anxious or more shy? Examining the social anxiety levels of adolescents with primary enuresis nocturna: a controlled study. J Pediatr Urol.

[18] Keten H, Ölmez S, Gençoğlan S (2014). Primer enürezis noktürna tanılı çocuk ve ergenlerde anksiyete ve depresyon belirti şiddetinin değerlendirilmesi. Ankara Med J.

[19] Van Hoecke E, Hoebeke P, Braet C, Walle JV (2004). An assessment of internalizing problems in children with enuresis. J Urol.

[20] Hjalmas K, Arnold T, Bower W (2004). Nocturnal enuresis: an international evidence based management strategy. J Urol.

[21] Iscan B, Ozkayın N (2020). Evaluation of health-related quality of life and affecting factors in child with enuresis. J Pediatr Urol.

[22] Joinson C, Heron J, Emond A, Butler R (2007). Psychological problems in children with bedwetting and combined (day and night) wetting: a UK population-based study. J Pediatr Psychol.

